# Health Promoting Compounds in Milk and Dairy Products

**DOI:** 10.3390/molecules31060919

**Published:** 2026-03-10

**Authors:** Mena Ritota, Pamela Manzi

**Affiliations:** Consiglio per la Ricerca in Agricoltura e l’Analisi dell’Economia Agraria, Centro di Ricerca Alimenti e Nutrizione, Via Ardeatina 546, 00178 Rome, Italy; pamela.manzi@crea.gov.it

## 1. Introduction

Milk represents a fundamental component of the human diet, as it supplies most of the nutrients essential for growth and development. It can be consumed in its natural or fermented form or processed into yoghurt, cheese, and a wide variety of other dairy products; it is also the basic ingredient for many products. Milk and dairy products are widely recognized as rich sources of calcium, which is present in a highly bioavailable form [1], but they also contribute significant amounts of specific minerals and vitamins [2].

The complex composition of milk, characterized by a wide range of nutrients and bioactive compounds and by the interactions among these components, positively influences digestion, nutrient absorption, and overall human health. This holistic perspective led the International Dairy Federation (IDF) in 2019 to introduce the concept of the “dairy matrix” [3]. Scientific evidence supporting this concept demonstrates that the health benefits of dairy products arise from the entire food matrix rather than from individual components alone. This dairy matrix exerts a wide range of effects, including antimicrobial, biostatic, antihypertensive, ACE-inhibitory, anticarcinogenic, anti-obesity, immunomodulatory, antidiabetic, cholesterol-lowering, probiotic, and prebiotic actions [4]. Consequently, the consumption of milk and dairy products goes beyond providing basic nutrition, as milk components exert relevant metabolic effects and contribute significantly to overall health.

The overall quality of milk and dairy products, as well as their content of health-promoting compounds, is affected by multiple aspects, including genetic factors [5], farming and feeding systems [6], technological processes (e.g., heat treatments [7], fermentation [8], or the addition of specific ingredients rich in bioactive compounds in the case of new product formulations [9], etc. For this reason, in recent years, emerging technologies—e.g., nanotechnology [10] and novel thermal and non-thermal processing [11]—have also been developed to provide new solutions for the incorporation of bioactive compounds into dairy products, enhancing the functional properties of these food items without compromising consumers’ sensory perception.

This Special Issue, entitled “Health-Promoting Compounds in Milk and Dairy Products”, was conceived to achieve the following aims: (i) explore recent advances in analytical methods for the assessment of bioactive compounds in dairy products; (ii) update current knowledge on the nutritional evaluation of milk-based foods; (iii) collect recent studies aimed at improving the nutritional value of milk and dairy products; and (iv) examine the effects of bioactive compounds from dairy products within the human diet.

Following a rigorous peer-review process, eight papers were accepted, including six original research articles, one review, and one opinion paper.

## 2. An Overview of the Published Articles

Lipids are key components of milk from both a nutritional and economic perspective. They supply a significant amount of energy and contribute to the sensory and physical properties of dairy products. In addition, milk fat acts as a vehicle for naturally occurring fat-soluble vitamins (A, D, E, and K) and β-carotene, a precursor of vitamin A [Contribution 1]. Milk fat is also naturally rich in saturated fatty acids (SFAs), and for this reason dairy fat is often perceived by consumers as increasing the risk of cardiovascular disease, metabolic syndrome, or obesity, leading to a preference for fat-free dairy products. Nonetheless, this negative perception is not supported by the most recent scientific evidence which suggests that dairy products could promote human health thanks to the presence of certain bioactive fatty acids [12]. For these reasons, accurate determination of the fatty acid profile of milk is of considerable importance. A range of analytical techniques is currently available for fatty acid analysis in food matrices, including high performance liquid chromatography (HPLC), gas chromatography (GC), near-infrared and mid-infrared spectroscopy (NIRS and MIRS, respectively), etc. Although conventional chemical methods such as HPLC and GC provide high analytical accuracy, they are often time-consuming, labor-intensive, and costly. In contrast, infrared spectroscopy-based approaches offer rapid and low-cost alternatives for predicting milk fatty acid composition [13], although further optimization is still required to enhance their predictive performance. Within this context, Zhao et al. [Contribution 1] evaluated the feasibility of using mid-infrared spectroscopy to predict the fatty acid composition of milk from Chinese Holstein cattle. The authors conducted a comprehensive comparison of four regression models, five spectral pre-processing methods, two spectral ranges, and two units of fatty acid expression (g/100 g milk and g/100 g fat). Among the tested approaches, random forest regression combined with derivative- and Savitzky–Golay-based preprocessing achieved the highest prediction accuracy. Seventeen fatty acids and fatty acid groups showed reliable predictive performance (R^2^ ≥ 0.75 and RPD ≥ 2), particularly when expressed on a milk basis. The study of Zhao et al. [Contribution 1] represents the first systematic MIRS-based evaluation of milk fatty acids in Chinese Holstein cows and highlights the potential of MIRS as a high-throughput, cost-effective phenotyping tool for genetic evaluation and dairy breeding programs.

Milk is widely recognized for the high nutritional quality of its protein, with caseins accounting for approximately 80% of the total protein fraction. Caseins are a heterogeneous group of proteins consisting of four polymorphisms: αS1-casein, the most abundant (38%), followed by β-casein (36%), κ-casein (13%) and αS2-casein (10%) [14]. β-caseins occur in several genetic variants, among which β-casein A1 and A2, differing by a single amino acid substitution at position 67, are the most common in European cattle breeds [15]. This structural difference is particularly relevant because β-casein A1 can release β-casomorphin-7 (BCM-7) during digestion, a bioactive peptide that has been associated with potential adverse health effects, including gastrointestinal disorders, type 1 diabetes, and coronary heart disease [16,17]. The health concerns related to β-casein A1 have led to increasing consumer demand for milk produced exclusively from A2A2 cows, which is marketed as a functional food and typically commands a premium price. Consequently, there is a strong need for analytical methods that are rapid, reliable, and sensitive enough to authenticate A2A2 milk and detect potential adulteration. As already mentioned above, conventional analytical techniques, such as Liquid Chromatography (LC), Enzyme Linked Immunosorbent Assay (ELISA), Polymerase Chain Reaction (PCR), Polyacrylamide Gel Electrophoresis (PAGE), restriction fragment length polymorphism (RFLP)-PCR, and Fourier-transform infrared (FTIR) spectroscopy, are often time-consuming, expensive, labor-intensive, or insufficiently sensitive, and many of them are unable to simultaneously distinguish and quantify β-casein A1 and A2 at the protein level [Contribution 2]. To address these limitations, Elferink et al. [Contribution 2] developed a competitive microsphere-based multiplex immunoassay capable of simultaneously detecting and distinguishing β-casein A1 and A2 in raw and pasteurized milk samples. Through optimized buffer conditions, the assay achieves <10% cross-reactivity between βCA1 and βCA2, demonstrating exceptional antibody specificity for nearly identical proteins. The assay detected adulteration of pure A2A2 milk with common bovine milk at levels as low as 1%, meeting and exceeding relevant regulatory sensitivity expectations. Beyond fraud detection, the method developed by the authors [Contribution 2] enables cow milk phenotyping directly from milk, providing a faster and less invasive alternative to DNA-based genotyping. Furthermore, compared to ELISA, PCR, and FTIR-based methods, the developed method is faster, simpler, has a semi-high throughput, and is well suited for routine quality control.

Regarding the mineral fraction, dairy products are widely recognized as excellent sources of calcium. However, the study by Manzi et al. [Contribution 3] on selected Italian cheeses showed that these products can also represent a valuable dietary source of zinc, an essential trace element involved in numerous catalytic, structural, and regulatory functions in the human body. The authors analyzed a total of 57 PDO (Protected Denomination of Origin) and Traditional Italian cheeses, reporting zinc concentrations ranging from 0.39 to 7.75 mg per 100 g of cheese. The considerable variability in zinc content observed among the samples was attributed to several factors, including feeding system and animal species (cow, sheep, goat, and buffalo), but it was mainly associated with the cheese-making process. Curdling and salting generally represent the two processing stages mainly affecting the mineral composition of cheese [18]. In addition, the authors [Contribution 3] assessed the nutritional relevance of zinc in the analyzed cheeses, showing that approximately half of the samples provided more than 20% of the European Daily Reference Intake for zinc (10 mg/day) [19] with a single serving of cheese (50 or 100 g), whereas only two samples contributed less than 4%, due to the acidic rather than enzymatic cheesemaking process. Overall, these findings support the role of cheese consumption as a valuable means of supplying dietary zinc.

Among fat-soluble vitamins, vitamin D plays a crucial role in the regulation of calcium metabolism and the maintenance of bone health [20,21]. Recent evidence also highlights its involvement in modulating immune system function [22,23]. In the absence of fortified foods, the contribution of the diet to the daily amount of vitamin D is generally scarce, and deficiencies of this vitamin have been reported in various population groups, including individuals living in regions with high sun exposure. Although the natural vitamin D content in cow’s milk is relatively low— typically ranging from 0.1 to 1 μg/L in whole milk [24,25]—vitamin D fortification of milk has proven to be an effective strategy for increasing population intake in many countries, such as in Canada, Finland, Sweden and Austria [26]. As a result, this practice is currently mandatory in some countries and strongly recommended in others [26]. In this context, Contribution 4 provides a comprehensive review of current evidence on vitamin D’s role in health and disease and explores how milk’s structural properties (particularly those related to the milk fat globule membrane and casein micelle organization), as well as industrial processing and storage conditions, impact vitamin D delivery. The authors emphasize the suitability of milk as an efficient vehicle for vitamin D, capable of supplying a modest yet nutritionally meaningful amount that contributes to achieving the adequate daily intake for this vitamin.

In the dairy sector, small-scale producers are increasingly focusing on innovation and high-quality products to meet consumer demand and enhance their market position. One common approach is the enrichment of cheeses with additional ingredients, such as colorants, spices, and flavoring agents, which can enhance sensorial, physical, chemical and nutritional quality of the final product. Saffron serves as a notable example of this practice. It is typically added into cheeses as a natural coloring agent but also contributes bioactive properties, due to the presence of crocins (water-soluble carotenoids responsible for saffron color and antioxidant activity), picrocrocin (responsible for saffron bitterness), and safranal (responsible for aroma and antioxidant potential). Ritota et al. [Contribution 5] were the first to evaluate saffron bioactive compounds in cheese by using a single UHPLC method and reported the first UHPLC-based assessment of safranal in cheese. Crocins were detected in all samples, with trans-4-GG identified as the predominant form, whereas picrocrocin was not detected, likely due to its degradation into safranal during cheesemaking. Safranal was found only in one ewe cheese with a high saffron content, probably because of its volatility, which makes it challenging to analyze accurately using liquid chromatography. Additionally, Contribution 5 assessed for the first time the antiproliferative effects of saffron within a food matrix, whereas prior studies had focused mainly on pure compounds (mostly crocin) or saffron extracts. The results showed no significant effect on CaCo2 cells (colon cancer model), while significant antiproliferative effects were observed on HeLa (cervix carcinoma) and MDA-MB-231 (breast cancer) cells. According to the authors [Contribution 5], the chemical environment of the cheese modulates the activity of saffron bioactive compounds, which is influenced not only by crocin concentration but also by residual protein/peptides and fat contents in the extracts. These findings support the concept of a “food matrix effect”, where the biological activity of compounds is modified by interactions within the entire food system. They also provide preliminary evidence that saffron-enriched cheeses may have functional food potential, thus opening opportunities for product innovation, added value for producers, and the development of functional dairy products.

The enrichment of dairy products with bioactive compounds was also investigated by Znamirowska et al. [Contribution 6], who added different sources of vitamin C (rosehip, acerola and ascorbic acid in powder form) to probiotic fermented milk to enhance its nutritional value and functionality. Compounds with antioxidant activity (phenolics and flavonoids) in rosehip and acerola were found to better protect vitamin C compared to pure ascorbic acid (in the synthetic form), highlighting a clear food matrix effect on vitamin C stability. The source of vitamin C (natural fruit matrix vs. synthetic) also had a significant impact on probiotic viability, product stability, texture, and sensory quality, with natural vitamin C sources enhancing overall sensory perception of the fermented milk. After 21 days of storage, *Lactobacillus rhamnosus* counts remained above 8 log CFU/g in all samples, exceeding the therapeutic minimum and confirming that all designed products met probiotic standards. Given the advantages of natural products and positive consumer perception, the authors [Contribution 6] suggest that rosehip and acerola are promising candidates for the development of functional dairy products for the market. This Contribution provides results with direct industrial applicability, offering practical guidance on formulation limits (maximum vitamin C dose without causing protein denaturation), effects on gel structure, storage stability data, and sensory acceptability confirmation.

Czarnowska-Kujawska and Paszczyk [Contribution 7] proposed the natural folate bio-fortification of milk through fermentation as a promising alternative to synthetic folic acid fortification, a common practice that can have drawbacks (e.g., masking vitamin B12 deficiency, potential risks of excessive intake). Fermented milk represents an ideal matrix for bio-fortification because it contains folate-binding proteins that enhance stability and bioavailability, is widely consumed, and can be enriched in situ through microbial metabolism. The authors [Contribution 7] evaluated folate production in fermented milk using different commercially available starter cultures, including *Streptococcus thermophilus*, *Lactobacillus delbrueckii* subsp. *Bulgaricus*, *Lactococcus lactis*, and *Bifidobacterium bifidum*. In addition to folate synthesis, these probiotic bacteria were assessed for their ability to synthesize conjugated linolenic acid *cis*9*trans*11 C18:2 (CLA). The results showed that mixed starter cultures were more effective than single cultures in producing folate, with the combination of *Lactobacillus* spp., *Streptococcus thermophilus*, and *Bifidobacterium bifidum* yielding the highest folate content (≈105 µg/kg) in fermented milk. Compared to pasteurized milk, folate levels increased 1.6–2.8 times in fermented products. However, microbial bio-fortification alone was insufficient to meet recommended daily folate intake, as the highest folate content would only cover approximately 25% of daily requirements if 1 kg of product were consumed. Furthermore, the authors highlighted that folate stability during storage was significantly influenced by starter culture composition, storage time, and the acidic environment, with losses reaching up to 65% in some formulations. Starter culture selection also impacted on the fatty acid profile, with fermented milks containing *Bifidobacterium bifidum* showing significant increases in CLA during storage, and CLA levels, like folate content, being affected by storage time. Contribution 7 represents one of the few studies combining vitamin bio-fortification and lipid functional enhancement in the same fermented dairy system. The findings strengthen the concept that functional dairy products should be designed through careful microbial consortia selection to maximize multiple health-promoting compounds while maintaining stability during shelf life but also highlight the need for further research to optimize conditions for producing effective functional dairy products.

Dabo et al. [Contribution 8] provided a comprehensive and critical overview of recent advances in whipping cream formulations, an oil-in-water emulsion widely used in the food industry and increasingly popular in the consumer market. The authors examined how structural and compositional parameters affect the performance, efficiency, and biological or chemical activity of whipping creams, while also addressing the main limitations of current approaches, including challenges related to stability, scalability, cost, and practical implementation. This analysis highlights the gap between laboratory-scale research and industrial or clinical translation.

In response to increasing consumer demand for healthier and higher-quality options, current and future research on whipping cream is focusing on the development of healthier products, including low-fat, low-sugar, functional whipping creams, and formulations enriched with probiotics. Another major trend is the development of plant-based alternatives to traditional dairy whipping cream. However, achieving sensory and functional properties comparable to conventional dairy products remains challenging, as plant proteins often require structural modifications (hydrolysis, particle formation, or microgel creation) and synergistic interactions with emulsifiers to replicate the performance of dairy proteins. Furthermore, a deeper understanding of the mechanisms governing low-molecular-weight surfactants and fat nucleation processes is essential to improve control over whipping cream structure and quality. Overall, Contribution 8 offers a valuable resource for researchers by consolidating dispersed knowledge, providing critical analysis, and presenting a forward-looking roadmap for future developments in the field.

## 3. Conclusions and Future Perspectives

This Special Issue highlights the central role of milk and dairy products as complex food systems in which nutritional value and health effects arise from the interaction among multiple components rather than from single nutrients alone. The concept of the dairy matrix clearly emerges across the collected contributions, reinforcing the idea that the structural organization of milk components (lipids, proteins, minerals, vitamins, and bioactive compounds) plays a crucial role in modulating digestion, bioavailability, and biological activity.

The published studies also reveal significant progress in both analytical and technological approaches. The development of rapid, reliable, and cost-effective analytical tools, such as mid-infrared spectroscopy and multiplex immunoassays, offers promising opportunities for high-throughput quality control, product authentication, and on-farm phenotyping, supporting innovation along the entire dairy value chain. At the same time, updated evidence on the mineral and vitamin composition of dairy products confirms their significant contribution to micronutrient intake, while simultaneously emphasizing how processing and formulation can enhance or limit their nutritional impact.

A recurring theme across several contributions is the enrichment and bio-fortification of dairy products with health-promoting compounds, achieved through the incorporation of natural ingredients, targeted fermentation strategies, or optimized fortification practices. These studies provide clear evidence that the food matrix plays a decisive role in determining compound stability, bioactivity, probiotic viability, and sensory acceptance. It is worth underlining that the development of functional dairy products must be approached holistically. This approach should balance nutritional improvement with considerations of technological feasibility, shelf-life stability, and consumer perception.

Looking forward, future research should focus on a deeper understanding of matrix–compound interactions, particularly during digestion and metabolism, in order to improve the prediction of health-related outcomes. Key research directions include the optimization of microbial consortia for multi-compound bio-fortification, the further development of non-thermal and emerging processing technologies, and the validation of functional effects through well-designed in vivo and clinical studies. Furthermore, bridging the gap between laboratory-scale innovation and industrial application remains a critical challenge in the design of new functional dairy products.

Overall, the contributions collected in this Special Issue confirm that milk and dairy products continue to be a dynamic and highly promising system for nutritional innovation. By combining advanced analytics, smart processing, and a matrix-oriented perspective, future developments in the dairy sector can lead to products that not only meet consumer expectations but also contribute meaningfully to public health and sustainable food systems.

The guest editors sincerely thank all the authors for the high quality of their contributions and extend their sincere appreciation to the reviewers for their insightful and constructive assessments. They also thank the *Molecules* editorial team for their professional guidance and support throughout the publication process. Hopefully, this Special Issue will serve as a valuable reference point and inspire further research into the field of health-promoting compounds in milk and dairy products.

## Data Availability

No new data were created or analyzed in this study. Data sharing is not applicable to this article.

## References

[1] Paggio F., Ritota M., Di Costanzo M.G., Barzaghi S., Monti L., Ulrici A., Manzi P. (2023). Effects of time and temperature of storage on chemical and nutritional characteristics of raw milk for Provolone Valpadana PDO cheesemaking: A multivariate approach. J. Dairy Res..

[2] Ritota M., Di Costanzo M.G., Rampanti G., Aquilanti L., De León C.M.B., Tejada L., Psomas A., Dridi B.A.M., Manzi P. (2025). Innovative thistle-curdled cheeses from the Mediterranean area: Nutritional evaluation of some relevant compounds. J. Dairy Res..

[3] International Dairy Federation (2023). Dairy Matrix: The Case of Milk (Factsheet of the IDF N° 33/2023). https://shop.fil-idf.org/products/factsheet-of-the-idf-n-33-2023-dairy-matrix-the-case-of-milk.

[4] Park Y.W. (2009). Bioactive Components in Milk and Dairy Products.

[5] Pilla F., Valentini A., Lenstra J.A., Martin P., Rubino R., Sepe L., Dimitriadou A., Gibon A. (2006). Animal genetics and functional food. Livestock Farming Systems.

[6] Morand-Fehr P., Fedele V., Decandia M., Le Frileux Y. (2007). Influence of farming and feeding systems on composition and quality of goat and sheep milk. Small Rumin. Res..

[7] Delorme M.M., Guimarães J.T., Coutinho N.M., Balthazar C.F., Rocha R.S., Silva R., Margalho L.P., Pimentel T.C., Silva M.C., Freitas M.Q. (2020). Ultraviolet radiation: An interesting technology to preserve quality and safety of milk and dairy foods. Trends Food Sci. Technol..

[8] Gemechu T. (2015). Review on lactic acid bacteria function in milk fermentation and preservation. Afr. J. Food Sci..

[9] Ritota M., Mattera M., Di Costanzo M.G., Manzi P. (2018). Evaluation of Crocins in Cheeses made with Saffron by UHPLC. J. Braz. Chem. Soc..

[10] Vélez M.A., Perotti M.C., Santiago L., Gennaro A.M., Hynes E., Mihai Grumezescu A. (2017). Bioactive compounds delivery using nanotechnology: Design and applications in dairy food. Nutrient Delivery.

[11] Ribeiro N.G., Xavier-Santos D., Campelo P.H., Guimarães J.T., Pimentel T.C., Duarte M.C.K., Freitas M.Q., Esmerino E.A., Silva M.C., Cruz A.G. (2022). Dairy foods and novel thermal and non-thermal processing: A bibliometric analysis. Innov. Food Sci. Emerg. Technol..

[12] Gómez-Cortés P., Juárez M., de la Fuente M.A. (2018). Milk fatty acids and potential health benefits: An updated vision. Trends Food Sci. Technol..

[13] Samková E., Špička J., Hanuš O., Roubal P., Pecová L., Hasoňová L., Smetana P., Klimešová M., Čítek J. (2020). Comparison of Fatty Acid Proportions Determined by Mid-Infrared Spectroscopy and Gas Chromatography in Bulk and Individual Milk Samples. Animals.

[14] De Kruif C.G., Huppertz T., Urban V.S., Petukhov A.V. (2012). Casein micelles and their internal structure. Adv. Colloid Interface Sci..

[15] Sebastiani C., Arcangeli C., Ciullo M., Torricelli M., Cinti G., Fisichella S., Biagetti M. (2020). Frequencies Evaluation of beta-Casein Gene Polymorphisms in Dairy Cows Reared in Central Italy. Animals.

[16] Jianqin S., Leiming X., Lu X., Yelland G.W., Ni J., Clarke A.J. (2016). Effects of milk containing only A2 beta casein versus milk containing both A1 and A2 beta casein proteins on gastrointestinal physiology, symptoms of discomfort, and cognitive behavior of people with self-reported intolerance to traditional cows’ milk. Nutr. J..

[17] McLachlan C.N.S. (2001). β-casein A1, ischaemic heart disease mortality, and other illnesses. Med. Hypotheses.

[18] Coni E., Bocca A., Ianni D., Caroli S. (1995). Preliminary evaluation of the factors influencing the trace element content of milk and dairy products. Food Chem..

[19] EU Regulation (EU) No 1169/2011 of the European Parliament and of the Council of 25 October 2011 on the Provision of Food Information to Consumers. https://eur-lex.europa.eu/legal-content/EN/ALL/?uri=CELEX%3A32011R1169.

[20] EFSA, Panel on Dietetic Products, Nutrition and Allergies (NDA) (2010). Scientific opinion on the substantiation of health claims related to vitamin D and normal function of the immune system and inflammatory response (ID 154, 159), maintenance of normal muscle function (ID 155) and maintenance of normal cardiovascular function (ID 159) pursuant to Article 13(1) of Regulation (EC) No 1924/2006. EFSA J..

[21] Institute of Medicine (2010). Dietary Reference Intakes for Calcium and Vitamin D.

[22] EFSA, Panel on Dietetic Products, Nutrition and Allergies (NDA) (2015). Scientific opinion on the substantiation of a health claim related to vitamin D and contribution to the normal function of the immune system pursuant to Article 14 of Regulation (EC) No 1924/2006. EFSA J..

[23] Scientific Advisory Committee on Nutrition (SACN) (2016). Vitamin D and Health Report Public Health England. https://www.gov.uk/government/publications/sacn-vitamin-d-and-health-report.

[24] Schmid A., Walther B. (2013). Natural Vitamin D Content in Animal Products. Adv. Nutr..

[25] Kasalová E., Aufartová J., Kujovská Krčmová L., Solichová D., Solich P. (2015). Recent trends in the analysis of vitamin D and its metabolites in milk—A review. Food Chem..

[26] Itkonen S., Erkkola M., Lamberg-Allardt C. (2018). Vitamin D Fortification of Fluid Milk Products and Their Contribution to Vitamin D Intake and Vitamin D Status in Observational Studies—A Review. Nutrients.

